# Comprehensive measurements of hydroxylinoleate and hydroxyarachidonate isomers in blood samples from primary open-angle glaucoma patients and controls

**DOI:** 10.1038/s41598-018-36952-6

**Published:** 2019-02-18

**Authors:** Aya Umeno, Masaki Tanito, Sachiko Kaidzu, Yasuyuki Takai, Masanori Horie, Yasukazu Yoshida

**Affiliations:** 1 Health Research Institute, National Institute of Advanced Industrial Science and Technology, 2217-14 Hayashicho, Takamatsu, Kagawa 761-0395 Japan; 2 Department of Ophthalmology, Shimane University Faculty of Medicine, 89-1 Enya, Izumo, Shimane 693-8501 Japan

## Abstract

We previously reported that lower systemic antioxidant capacity is involved in primary open-angle glaucoma (POAG) and exfoliation syndrome pathogeneses as measured by ferric-reducing activity. In the present study, we measured hydroxylinoleate (HODE) and hydroxyarachidonate (HETE) isomer serum levels after sample reduction and saponification to investigate POAG pathogenesis. POAG patients (n = 198) were recruited and divided into normal- and high-tension glaucoma groups (n = 84 and 114, respectively) depending on intraocular pressure. Total HODE (/linoleic acid) and HETE (/arachidonic acid) serum levels were significantly higher in the POAG group (211.9 ± 143.0 and 181.0 ± 164.1 µmol/mol, respectively) than in controls (167.1 ± 105.2 and 132.5 ± 139.7 µmol/mol, p = 0.0025 and 0.0101, respectively). The associations between HODEs/HETEs and glaucoma were further confirmed by multivariate analyses after adjusting for differences in demographic parameters. Among the HODE isomers, the levels of 9- and 13-(Z,E)-HODEs (p = 0.0014) and singlet oxygen-specific products (i.e., 10- and 12-(Z,E)-HODEs, p = 0.0345) were higher in the POAG group than in controls, while free radical-mediated oxidation-specific products (i.e., 9- and 13-(E,E)-HODEs, p = 0.0557) demonstrated a marginal difference. Enzymatic and singlet oxygen-mediated fatty acid oxidation may be major pathways of oxidation process in glaucoma subjects.

## Introduction

**Figure 1:**
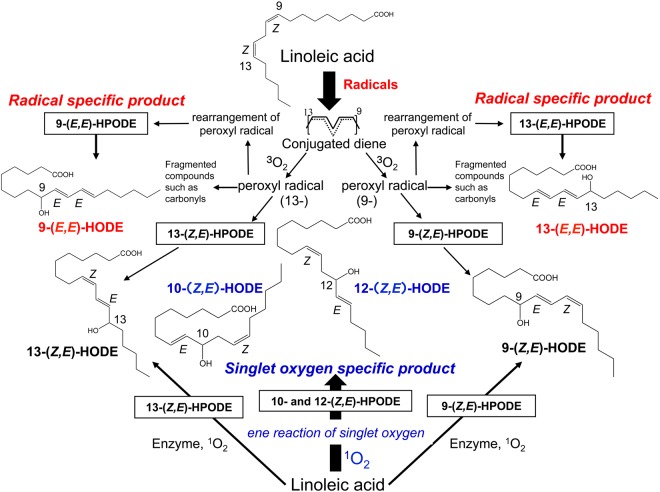
Chemical structures of HODE isomers.

We proposed totally assessed hydroxylinoleates (HODEs) from biological samples as biomarkers for investigations of pathogenesis, disease progression, and prognosis^1–4^. Furthermore, they can provide information on the effectiveness of functional factors in foods for the prevention of diseases. The advantage of measuring oxidation products of linoleates is that they are the most abundant polyunsaturated fatty acids *in vivo* and their oxidation proceeds by a straightforward mechanism that yields much simpler products than arachidonates and more highly unsaturated fatty acids such as docosahexaenoates^5^. Hydroperoxy octadecadienoates (HPODEs), derived from linoleates, are primary products obtained by simple mechanisms from abundant parent lipids *in vivo*. The absolute concentrations of lipid hydroperoxides *in vivo* are considered minimal since they are substrates of many enzymes such as glutathione peroxidases and phospholipases. In such cases, the stable oxidation products are HODEs. Our method for measuring totally assessed HODE is characterized by the reduction and saponification of samples beforehand. By this method, we can measure the ester and free forms of hydroperoxides and hydroxides as HODE. H(P)ODEs are formed by a radical-mediated oxidation mechanism that consists of four isomers (Fig. 1): 13-(Z,E)-H(P)ODE, 13-(E,E)-H(P)ODE, 9-(E,Z)-H(P)ODE, and 9-(E,E)-H(P)ODE. Little 11-H(P)ODE is formed under normal conditions as the pentadienyl radical that is formed by the abstraction of hydrogen at the 11-carbon, which rearranges rapidly to form stable conjugated diene radicals. Some other by-products of these radicals are known as ketones. 9- and 13-(Z,E)-H(P)ODE are also formed by enzymatic oxidation via lipoxygenase as enantio-, regio-, and stereo-specific products^6^. Thus, 9- and 13-(E,E)-H(P)ODE are specific products of radical-mediated oxidation. On the other hand, singlet oxygen oxidizes linoleic acids (LAs) by non-radical oxidation to form 13-(Z,E)-H(P)ODE, 10-(E,Z)-H(P)ODE, 12-(Z,E)-H(P)ODE, and 9-(E,Z)-H(P)ODE. In this case, 10- and 12-(Z,E)-H(P)ODEs are specific oxidation products of singlet oxygen^7^. There are some reports that describe the formation of singlet oxygen *in vivo*. For example, singlet oxygen is formed by the Russell mechanism, the coupling of peroxyl radicals, and from the reaction of hydroperoxides with metal ions, HOCl, peroxynitrite, and cytochrome c^8^. Other works showed Type I and Type II photooxidation mechanisms in the presence of photosensitizers^9–13^. The implications of using HODEs and hydroxyarachidonates (HETEs) as biomarkers for detecting early-stage diseases were reviewed previously^6^. Among them, the usefulness for detecting the risk of diabetes is striking^7,14^.

Glaucoma is characterized as progressive glaucomatous optic neuropathy leading to visual field loss. It is a cause of irreversible blindness worldwide^15–17^. The death of retinal ganglion cells (RGCs) and RGC axon loss cause glaucomatous optic neuropathy, in which elevated intraocular pressure (IOP) is the primary risk factor^15^. The IOP in patients with primary open-angle glaucoma (POAG) increases because of reduced aqueous humour outflow at the trabecular meshwork (TM)^18^. Treatment using hydrogen peroxide affects the cytoskeletal structure and cell-matrix interactions in TM cells^19^; depletion of glutathione and hydrogen peroxide treatment decrease TM outflow facility^20^. Various oxidative stresses have been reported to induce RGC death in experimental studies^21,22^; free-radical scavengers prevent glaucomatous tissue injury, specifically, glutamate- and IOP-induced RGC death^23,24^. Evidence suggests that oxidative stress is involved in IOP elevation and RGC loss in POAG and in POAG without marked IOP elevation such as in normal-tension glaucoma (NTG). We previously reported lower systemic antioxidant capacity measured by ferric-reducing activity in patients with POAG and glaucoma secondary to exfoliation syndrome compared with controls^17^. We also have shown that lower systemic antioxidant capacity measured by ferric-reducing activity was associated with higher IOP and more severe visual field damage in open-angle glaucoma in a comprehensive assessment^25,26^. In the present study, we investigated the involvement of oxidative stress in this disease by using our own proposed oxidative stress marker HODEs and HETEs quantitatively.

## Results

### Demographic data

Table 1 shows the demographic data of the subjects in this study. As shown, the IOP on the blood sampling day and highest IOP recorded in the POAG group were significantly higher than those in the control (CT) group (p < 0.0492 and p < 0.0001, respectively). We divided the POAG group into the NTG and high-tension glaucoma (HTG) groups depending on the IOP. The IOP on the blood sampling day and highest IOP recorded in the HTG group were significantly higher than those in the CT and NTG groups (p < 0.0001 for all comparison pairs). Other than these factors, age, sex, diastolic blood pressure (DBP), and diabetes status were different in at least one pair of comparisons among the CT, NTG, and HTG groups.

**Table 1:** Demographic subjects data.

Parameters	CT	POAG	p-value^a^			NTG	HTG	
n	119	198				84	114	p-value^b^
Age (yrs)			0.8653					0.0470^*^
Mean ± SD	70.6 ± 10.9	70.4 ± 11.1				72.4 ± 10.7	69.0 ± 11.2	
range	23.0–92.0	24.0–89.0				25.0–89.0	24.0–88.0	
				*p-value*, v.s. CT^c^		0.0988	0.2881	
				*p-value*, v.s. NTG^c^			0.0172	
SEX			0.0046^**^					0.0059^**^
Men, n (%)	36 (30.3)	92 (46.5)				34 (40.5)	58 (50.9)	
Women, n (%)	83 (69.7)	106 (53.5)				50 (59.5)	56 (49.1)	
				*p-value*, v.s. CT^c^		0.1372	0.0014^##^	
				*p-value*, v.s. NTG^c^			0.1530	
SBP (mmHg)			0.0902					0.2372
Mean ± SD	135.3 ± 17.9	139.9 ± 20.3				139.5 ± 19.8	140.1 ± 20.7	
range	94.0–179.0	89.0–211.0				90.0–182.0	89.0–211.0	
DBP (mmHg)			0.0604					0.0286^*^
Mean ± SD	74.0 ± 11.1	77.1 ± 12.9				74.8 ± 11.2	78.8 ± 13.8	
range	45.0–98.0	47.0–133.0				47.0–100.0	51.0–133.0	
				*p-value*, v.s. CT^c^		0.6464	0.0118^#^	
				*p-value*, v.s. NTG^c^			0.0546	
Pulse rate (/min.)			0.1426					0.0777
Mean ± SD	73.0 ± 12.0	75.6 ± 14.7				73.7 ± 14.3	77.0 ± 14.8	
range	46.0–109.0	42.0–131.0				51.0–114.0	42.0–131.0	
Duration from the last meal (h)			0.2574					0.5176
Mean ± SD	3.9 ± 2.7	3.8 ± 2.1				3.7 ± 1.3	3.9 ± 2.5	
range	1.0–18.0	1.0–19.0				1.5–8.0	1.0–19.0	
Diabetes			0.0853					0.0315^*^
Yes, n (%)	18 (15.1)	46 (23.2)				14 (16.7)	32 (28.1)	
No, n (%)	101 (84.9)	152 (76.8)				70 (83.3)	82 (71.9)	
				*p-value*, v.s. CT^c^		0.8456	0.0173	
				*p-value*, v.s. NTG^c^			0.0638	
Current smoking habit			0.4651					0.1640
Yes, n (%)	11 (9.2)	25 (12.7)				7(8.3)	18 (15.9)	
No, n (%)	108 (90.8)	172 (87.3)				77 (91.7)	95 (84.1)	
IOP on blood sampling day (mmHg)			0.0492^*^					<0.0001^**^
Mean ± SD	13.8 ± 3.0	15.6 ± 6.0				13.4 ± 2.8	17.3 ± 7.1	
range	6.0–21.0	5.0–52.0				6.0–23.0	5.0–52.0	
				*p-value*, v.s. CT^c^		0.1585	<0.0001^##^	
				*p-value*, v.s. NTG^c^			<0.0001^##^	
Highest IOP recorded (mmHg)			<0.0001^**^					<0.0001^**^
Mean ± SD	16.7 ± 3.7	21.7 ± 8.3				17.2 ± 6.0	25.0 ± 8.2	
range	9.0–36.0	10.0–58.0				10.0–58.0	13.0–56.0	
				*p-value*, v.s. CT^c^		0.9854	<0.0001^##^	
				*p-value*, v.s. NTG^c^			<0.0001^##^	

^a^Comparison between CT and POAG groups by using Mann-Whitney U test for continuous data and by using Fisher’s exact probability test for categorical data. The ^*^and ^**^correspond to the significance levels at 5% (P < 0.05) and 1% (P < 0.01), respectively.

^b^Comparison among CT, NTG, and HTG groups by using Kruskal Wallis Test for continuous data and by using G test for categorical data. The ^*^and ^**^correspond to the significance levels at 5% (P < 0.05) and 1% (P < 0.01), respectively.

^c^Comparison between either pairs of CT, NTG, or HTG groups by using post-hoc Mann-Whitney U test for continuous data and by using Fisher’s exact probability test for categorical data. The ^#^and ^##^correspond to the significance levels at 5% (P < 0.0167) and 1% (P < 0.0033), respectively, by Bonferroni correction for multiple comparisons.

CT, control; POAG, primary open angle glaucoma; HTG, high tension glaucoma; NTG, normal tension glaucoma; SBP, systolic blood pressure; DBP, diastolic blood pressure; IOP, intraocular pressure.

### Serum levels of HODEs and HETEs in the CT and POAG groups

**Figure 2:**
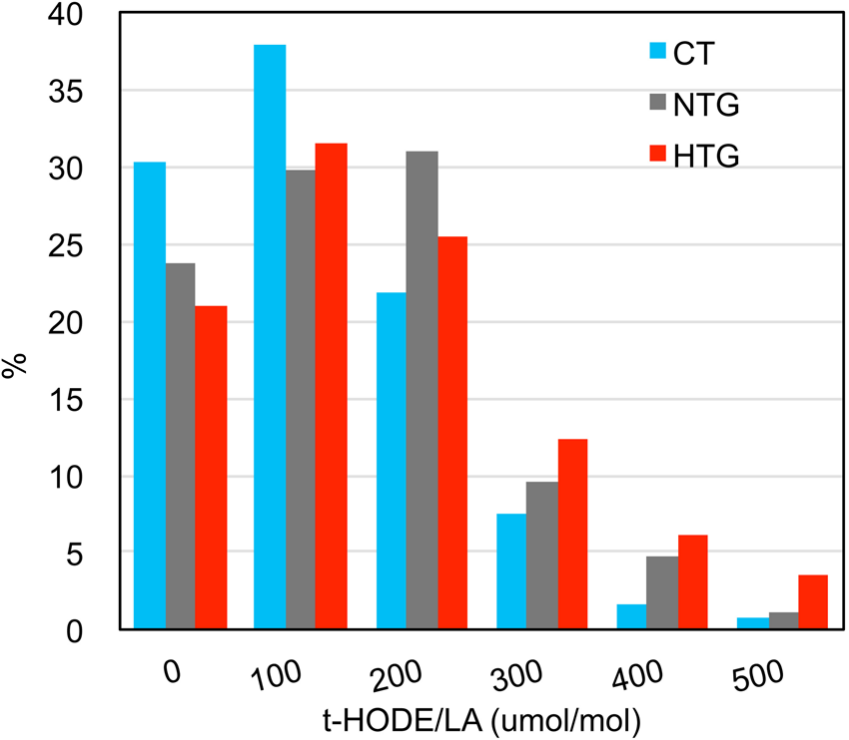
Distributions of t-HODE/LA levels in CT, NTG, and HTG subjects.

We measured serum levels of oxidative stress markers, HODEs, and HETEs together with their parent molecules, LAs and arachidonic acids (AAs), respectively, especially focusing on the isomer distributions. All results are summarized in Table 2. As shown, the total HODE (t-HODE) and total HETE (t-HETE) serum levels in the POAG group were significantly higher than those in the CT group (p = 0.0025 and 0.0101, respectively). For each HODE isomer, 9- and 13-(Z,E)-HODEs/LA, which are yielded by enzymatic, free radical, and singlet oxygen-mediated oxidation, demonstrated higher levels in the POAG group than in the CT group (p = 0.0014–0.0041). On the other hand, the 9- and 13-(E,E)-HODE/LA, which are yielded by free radical-mediated oxidation, were only marginally or non-significantly different between the POAG and CT groups (p = 0.0381–0.0874). The 10- and 12-(Z,E)-HODEs, which are singlet oxygen-specific products, were significantly higher in the POAG group than in the CT group (p = 0.0238–0.0345). An important observation is that the levels of 9- and 13-(Z,E)-HODEs/LA were one hundred times higher than those of 10- and 12-(Z,E)-HODEs/LA in both the CT and POAG groups. Singlet oxygen results in equal amounts of 9-, 10-, 12-, and 13-(Z,E)-HODEs. These data suggest that the dramatic increases in the levels of the 9- and 13-(Z,E)-HODEs/LA in the POAG group compared with the CT group were due to enzymatic oxidation, which is usually attributed to adaptive oxidation yielding physiological compounds such as prostaglandin. The levels of 5-, 12-, and 15-HETEs/AA in the POAG group were significantly higher than those in the CT group (p = 0.0051–0.0166) although this observation does not lead to a mechanistic discussion since these HETEs are formed by three common mechanisms of oxidation. In the subgroup analysis among the CT, NTG, and HTG groups, all oxidation markers showed significant differences between the HTG and CT groups, while no oxidation marker showed a difference between the NTG and CT groups. Distributions of t-HODE/LA levels in the CT, NTG, and HTG groups are shown in Fig. 2. As shown, among the groups, levels are highest in the HTG group in subjects with t-HODE/LA levels exceeding 300 µmol/mol.

**Table 2:** Comparison of serum HODEs/HETEs levels among groups.

Parameters	CT	POAG	p-value^a^	NTG	HTG	p-value^b^
n	119	198		84	114	
9-(*Z*,*E*)-HODE/LA (µmol/mol)			0.0041^**^			0.0103^*^
Mean ± SD	79.8 ± 54.9	103.1 ± 77.1		94.7 ± 60.4	109.3 ± 87.2	
range	12.4–296.2	11.8–665.7		12.2–260.8	11.8–665.7	
			*p-value*, v.s. CT^c^	0.0734	0.0029^##^	
			*p-value*, v.s. NTG ^c^		0.3378	
9-(*E*,*E*)-HODE/LA (µmol/mol)			0.0874			0.1628
Mean ± SD	16.4 ± 16.2	17.9 ± 16.9		16.5 ± 15.3	18.9 ± 18.0	
range	2.8–83.3	2.2–124.8		2.2–80.7	2.3–127.8	
10-(*Z*,*E*)-HODE/LA (µmol/mol)			0.0794			0.0553
Mean ± SD	0.8 ± 0.9	0.9 ± 0.7		0.8 ± 0.6	1.0 ± 0.8	
range	0.1–9.0	0.0–6.4		0.0–3.7	0.1–6.4	
12-(*Z*,*E*)-HODE/LA (µmol/mol)			0.0238^*^			0.0069^**^
Mean ± SD	0.8 ± 0.9	0.9 ± 0.7		0.7 ± 0.6	0.9 ± 0.8	
range	0.1–9.5	0.0–7.7		0.0–3.6	0.0–7.7	
			*p-value*, v.s. CT^c^	0.6207	0.0021^##^	
			*p-value*, v.s. NTG ^c^		0.0335	
10- and 12-(*Z*,*E*)-HODE/LA ^A^ (µmol/mol)			0.0345^*^			0.0140^*^
Mean ± SD	1.6 ± 1.8	1.8 ± 1.4		1.6 ± 1.1	1.9 ± 1.6	
range	0.2–18.5	0.1–14.1		0.1–7.3	0.2–14.1	
			*p-value*, v.s. CT^c^	0.5902	0.0049^#^	
			*p-value*, v.s. NTG ^c^		0.0447	
13-(*Z*,*E*)-HODE/LA (µmol/mol)			0.0014^**^			0.0027^*^
Mea ± SD	57.7 ± 42.5	76.0 ± 55.6		69.8 ± 47.8	80.6 ± 60.5	
range	9.6–232.0	6.8–437.4		6.8–199.5	8.6–437.4	
			*p-value*, v.s. CT^c^	0.0645	0.0006^##^	
			*p-value*, v.s. NTG ^c^		0.2015	
13-(*E*,*E*)-HODE/LA (µmol/mol)			0.0381^*^			0.0663
Mean ± SD	13.3 ± 12.1	14.9 ± 13.0		13.7 ± 11.9	15.8 ± 13.7	
range	2.8–53.7	2.2–83.6		2.2–58.1	2.9–83.6	
9- and 13-(*Z*,*E*)-HODE/LA ^B^ (µmol/mol)			0.0026^**^			0.0057^**^
Mean ± SD	137.4 ± 97.0	179.1 ± 130.8		164.5 ± 107.7	189.9 ± 144.9	
range	22.5–528.2	19.0–1103.1		19.0–460.3	20.4–1103.1	
			*p-value*, v.s. CT^c^	0.0703	0.0015^##^	
			*p-value*, v.s. NTG ^c^		0.2609	
9- and 13-(*E*,*E*)-HODE/LA^C^ (µmol/mol)			0.0557			0.1041
Mean ± SD	29.7 ± 28.1	32.8 ± 29.6		30.2 ± 27.1	34.7 ± 31.2	
range	6.8–132.1	4.4–208.4		4.4–138.8	5.3–208.4	
t-HODE^(A+B+C)^ (µmol/mol)			0.0025^**^			0.0052^**^
Mean ± SD	167.1 ± 105.3	211.9 ± 143.0		194.7 ± 113.3	224.5 ± 160.7	
range	29.3–572.1	25.7–1311.5		28.3–502.0	25.7–1311.5	
			*p-value*, v.s. CT^c^	0.0742	0.0013^##^	
			*p-value*, v.s. NTG ^c^		0.2362	
ZE/EE ratio ^(B^/^C)^			0.0884			0.2228
Mean ± SD	7.0 ± 6.2	7.7 ± 5.4		7.6 ± 5.5	7.8 ± 5.3	
range	0.9–36.3	0.9–25.8		1.0–25.8	0.9–24.8	
5-HETE/AA ^D^ (µmol/mol)			0.0051^**^			0.0117^*^
Mean ± SD	51.1 ± 50.8	69.0 ± 56.4		63.8 ± 54.3	72.8 ± 57.8	
range	2.2–280.2	1.1–242.1		2.0–218.0	1.1–240.1	
			*p-value*, v.s. CT^c^	0.0973	0.0029^##^	
			*p-value*, v.s. NTG ^c^		0.3155	
12-HETE/AA ^E^ (µmol/mol)			0.0159^*^			0.0403^*^
Mean ± SD	44.5 ± 52.5	59.8 ± 74.5		53.4 ± 51.5	64.6 ± 87.6	
range	0.2–263.2	0.1–800.6		0.1–228.5	0.3–800.6	
			*p-value*, v.s. CT^c^	0.1345	0.0123^#^	
			*p-value*, v.s. NTG ^c^		0.4381	
15-HETE/AA^F^ (µmol/mol)			0.0166^*^			0.0217^*^
Mean ± SD	37.0 ± 40.9	52.1 ± 51.6		47.7 ± 50.3	55.4 ± 52.5	
range	0.3–220.6	0.5–267.0		0.5–267.0	0.8–224.8	
			*p-value*, v.s. CT^c^	0.2645	0.0053^#^	
			*p-value*, v.s. NTG ^c^		0.1754	
t-HETE^(D+E+F)^(µmol/mol)			0.0101^*^			0.0202^*^
Mean ± SD	132.5 ± 139.7	181.0 ± 164.1		164.9 ± 148.5	192.8 ± 174.3	
range	3.5–764.0	3.2–1070.2		4.8–607.0	3.2–1070.2	
			*p-value*, v.s. CT^c^	0.1602	0.0047^#^	
			*p-value*, v.s. NTG ^c^		0.3035	

^a^Comparison between CT and POAG groups by using Mann-Whitney U test. The ^*^and ^**^correspond to the significance levels at 5% (P < 0.05) and 1% (P < 0.01), respectively.

^b^Comparison among CT, NTG, and HTG groups by using Kruskal Wallis Test. The ^*^and ^**^correspond to the significance levels at 5% (P < 0.05) and 1% (P < 0.01), respectively.

^c^Comparison between either pairs of CT, NTG, or HTG groups by using post-hoc Mann-Whitney U test. The ^#^and ^##^correspond to the significance levels at 5% (P < 0.0167) and 1% (P < 0.0033), respectively, by Bonferroni correction for multiple comparisons.

CT, control; POAG, primary open angle glaucoma; HTG, high tension glaucoma; NTG, normal tension glaucoma; HODE, hydroxylinoleates, LA, linoleic acid; t-HODE, total HODE; HETE, hydroxyarachidonates; AA, arachidonic acid; t-HETE, total HETE.

### Possible correlations between systemic HODEs/HETEs and clinical parameters

We further analysed correlations between oxidation markers and clinical parameters to specify oxidation mechanisms. Among the various demographic parameters, we selected age, sex, diabetes status, DBP, and IOPs (IOP on the blood sampling day and the highest IOP recorded) for analyses since these items were different between the CT and glaucoma groups (Table 3). In the univariate analyses, as shown in Table 3, a young age, the male sex, the absence of diabetes, a higher DBP, and higher IOPs were associated with higher HODEs/HETEs. It seems characteristic that the 10- and 12-(Z,E)-HODEs were associated with the IOPs with the highest significance among the various HODEs/HETEs. After adjustment for differences in demographic parameters between the CT and POAG groups by multiple regression analyses, as shown in Table 4, the highest IOP recorded and 9-(Z,E)-HODE, 13-(Z,E)-HODE, t-HODE, 15-HETE, and t-HETE were associated with the presence of glaucoma, whereas the effects of age, sex, diabetes status, and DBP disappeared.

**Table 3:** Correlation between serum HODEs/HETEs levels and demographic subjects data.

	Age (yrs)		SEX (men = 0, women = 1)		Diabetes (no = 0, yes = 1)		DBP (mmHg)		IOP on blood sampling day (mmHg)		Highest IOP recorded (mmHg)	
	*R*	*p-value*	*R*	*p-value*	*R*	*p-value*	*R*	*p-value*	*R*	*p-value*	*R*	*p-value*
9-(Z,E)-HODE/LA	−0.233	<0.0001^**^	−0.180	0.0013^**^	−0.188	0.0008^**^	0.147	0.0086^**^	0.080	0.1570	0.093	0.0992
9-(E,E)-HODE/LA	−0.144	0.0100^*^	−0.157	0.0049^**^	−0.027	0.6308	0.124	0.0273^*^	0.072	0.2033	0.080	0.1577
10-(Z,E)-HODE/LA	−0.236	<0.0001^**^	−0.154	0.0058^**^	−0.080	0.1565	0.097	0.0855	0.091	0.1066	0.137	0.0143^*^
12-(Z,E)-HODE/LA	−0.190	0.0010^**^	−0.262	<0.0001^**^	−0.086	0.1252	0.175	0.0017^**^	0.173	0.0020^**^	0.186	0.0009^**^
10- and 12-(Z,E)- HODE/LA^A^	−0.232	<0.0001^**^	−0.227	<0.0001^**^	−0.084	0.1337	0.152	0.0066^**^	0.135	0.0160^*^	0.166	0.0031^**^
13-(Z,E)-HODE/LA	−0.257	<0.0001^**^	−0.185	0.0010^**^	−0.181	0.0012^**^	0.170	0.0023^**^	0.097	0.0844	0.100	0.0755
13-(E,E)-HODE/LA	−0.165	0.0030^**^	−0.178	0.0015^**^	−0.031	0.5824	0.160	0.0044^**^	0.097	0.0847	0.090	0.1108
9- and 13-(Z,E)-HODE/LA^B^	−0.245	<0.0001^**^	−0.181	0.0012^**^	−0.185	0.0009^**^	0.159	0.0046^**^	0.088	0.1169	0.095	0.0909
9- and 13-(E,E)-HODE/LA^C^	−0.152	0.0070^**^	−0.170	0.0024^**^	−0.027	0.6270	0.142	0.0116^*^	0.085	0.1301	0.085	0.1324
t-HODE ^(A+B+C)^	−0.263	<0.0001^**^	−0.208	0.0002^**^	−0.169	0.0025^**^	0.177	0.0016^**^	0.087	0.1201	0.092	0.1017
ZE/EE^(B/C)^	−0.100	0.0750	−0.029	0.6098	−0.159	0.0045^**^	0.048	0.3972	0.005	0.9326	0.015	0.7921
5-HETE/AA^D^	−0.192	0.0010^**^	−0.143	0.0108^*^	−0.172	0.0021^**^	0.113	0.0453^*^	0.088	0.1175	0.103	0.0680
12-HETE/AA^E^	−0.216	<0.0001^**^	−0.152	0.0066^**^	−0.183	0.0011^**^	0.153	0.0063^**^	0.131	0.0194^*^	0.118	0.0358^*^
15-HETE/AA^F^	−0.241	<0.0001^**^	−0.156	0.0055^**^	−0.160	0.0042^**^	0.147	0.0087^**^	0.087	0.1206	0.094	0.0962
t-HETE^(D+E+F)^	−0.215	<0.0001^**^	−0.147	0.0087^**^	−0.171	0.0022^**^	0.140	0.0127^*^	0.114	0.0431^*^	0.108	0.0538

The correlation coefficient (r) and P values are calculated by Spearman’s rank correlation test. ^*^P < 0.05, ^**^P < 0.01.

**Table 4:** The multiple logistic regression analysis for Age, SEX, Diabetes, DBP, IOP, Highest IOP recorded and each lipid oxidation stress marker.

		Age	SEX	DBP	Diabetes	IOP	Highest IOP recorded	9-(*Z*,*E*)-HODE/LA	9-(*E*,*E*)-HODE/LA	10-and 12-(*Z*,*E*)/LA	13-(*Z*,*E*)-HODE/LA	13-(*E*,*E*)-HODE/LA
		(yrs)		(mmHg)		(mmHg)	(mmHg)	(μmol/mol)	(μmol/mol)	(μmol/mol)	(μmol/mol)	(μmol/mol)
P-value		0.2407	0.3937	0.2601	0.3121	0.1806	0.0000^**^	0.0116^**^				
Odds Ratio		1.0147	0.7838	1.0133	1.4358	0.9366	1.2247	1.0059				
95% CI range	Min-Max	0.99–1.04	0.45–1.37	0.99–1.04	0.71–2.90	0.85–1.03	1.13–1.33	1.00–1.01				
p-value		0.4035	0.2100	0.2695	0.5725	0.2203	0.0000^**^		0.5954			
Odds Ratio		1.0103	0.7043	1.0128	1.2163	0.9422	1.2227		1.0042			
95% CI range	Min-Max	0.99–1.03	0.41–1.22	0.99–1.04	0.62–2.40	0.86–1.04	1.13–1.33		0.99–1.02			
p-value		0.4085	0.2280	0.2494	0.5541	0.2213	0.0000^**^			0.6174		
Odds Ratio		1.0102	0.7112	1.0134	1.2288	0.9423	1.2215			1.0444		
95% CI range	Min-Max	0.99–1.03	0.41–1.24	0.99–1.04	0.62–2.43	0.86–1.04	1.12–1.33			0.88–1.24		
p-value		0.1999	0.3682	0.2631	0.2906	0.1591	0.0000^**^				0.0048^**^	
Odds Ratio		1.0163	0.7734	1.0132	1.4581	0.9332	1.2279				1.0086	
95% CI range	Min-Max	0.99–1.04	0.44–1.35	0.99–1.04	0.72–2.93	0.85–1.03	1.13–1.34				1.00–1.01	
p-value		0.3802	0.2122	0.2739	0.5620	0.2179	0.0000^**^					0.4302
Odds Ratio		1.0108	0.7060	1.0127	1.2230	0.9419	1.2226					1.0084
95% CI range	Min-Max	0.99–1.04	0.41–1.22	0.99–1.04	0.62–2.41	0.86–1.04	1.13–1.33					0.99–1.03
		Age	SEX	DBP	Diabetes	IOP	Highest IOP recorded	5-HETE/AA	12-HETE/AA	15-HETE/AA	t-HODE/LA	t-HETE/AA
		(yrs)		(mmHg)		(mmHg)	(mmHg)	(μmol/mol)	(μmol/mol)	(μmol/mol)	(μmol/mol)	(μmol/mol)
p-value		0.2447	0.3224	0.2746	0.3340	0.1681	0.0000^**^	0.0109^*^				
Odds Ratio		1.0147	0.7551	1.0129	1.4073	0.9347	1.2276	1.0066				
95%CI range	Min-Max	0.99–1.04	0.43–1.32	0.99–1.04	0.70–2.81	0.85–1.03	1.13–1.34	1.00–1.01				
p-value		0.3462	0.2580	0.2901	0.4667	0.1803	0.0000^**^		0.1583			
Odds Ratio		1.0117	0.7275	1.0123	1.2900	0.9366	1.2252		1.0037			
95% CI range	Min-Max	0.99–1.04	0.42–1.26	0.99–1.04	0.65–2.56	0.85–1.03	1.13–1.33		1.00–1.01			
p-value		0.2425	0.3121	0.2572	0.4084	0.1670	0.0000^**^			0.0191^*^		
Odds Ratio		1.0148	0.7511	1.0134	1.3355	0.9344	1.2280			1.0070		
95% CI range	Min-Max	0.99–1.04	0.43–1.31	0.99–1.04	0.67–2.65	0.85–1.03	1.13–1.34			1.00–1.01		
p-value		0.2037	0.4132	0.2798	0.2996	0.1731	0.0000^**^				0.0078^**^	
Odds Ratio		1.0161	0.7912	1.0127	1.4503	0.9355	1.2265				1.0033	
95% CI range	Min-Max	0.99–1.04	0.45–1.39	0.99–1.04	0.72–2.93	0.85–1.03	1.13–1.33				1.00–1.01	
p-value		0.2643	0.3092	0.2800	0.3829	0.1641	0.0000^**^					0.0260^*^
Odds Ratio		1.0140	0.7498	1.0127	1.3591	0.9341	1.2275					1.0021
95% CI range	Min-Max	0.99–1.04	0.43–1.31	0.99–1.04	0.68–2.71	0.85–1.03	1.13–1.33					1.00–1.00

IOP; IOP on blood sampling day.

## Discussion

Our method to measure HODE isomers is to assess them directly by high-performance liquid chromatography tandem mass spectrometry (HPLC-MS/MS) after saponification and reduction of the biological samples. Using this protocol, large amounts of oxidation products from linoleates are quantitatively assessed. The advantage of measuring HODEs as oxidative stress markers is that we can understand the oxidative mechanism in diseases by the distribution of their isomers. The standardization of their absolute values by their parent molecules’ linoleates is another advantage for comparisons among individuals. As shown in Fig. 1, four isomers of 9- and 13-(Z,E) and (E,E)-HODEs are yielded by free radical-mediated oxidation from the reduction of corresponding hydroperoxides (HPODEs)^6^. Singlet oxygen also produces four isomers of 9-, 10-, 12-, and 13-(Z,E)-HODEs by the reduction of the corresponding HPODEs. Only enzymes such as 15-lipoxygenase are known to yield 9- and 13-(Z,E)-HPODEs in *in vivo* enzymatic oxidation leading to the corresponding 9- and 13-(Z,E)-HODEs. Accordingly, 10- and 12-(Z,E)-HODEs are specific products from singlet oxygen while 9- and 13-(E,E)-HODEs are free radical-specific ones. The amounts from the subtraction of 9- and 13-(E,E)-HODEs and 10- and 12-(Z,E)-HODEs from 9- and 13-(Z,E)-HODEs are attributed to enzymatic oxidation *in vivo*. This study (Table 2) together with others suggest that singlet oxygen-mediated oxidation was minimal at less than one-fold in magnitude *in vivo*; especially, measured plasma levels and the levels of free radical-mediated oxidation were also lower than those of enzymatic oxidation.

We found that several HODEs/HETE were higher in the POAG group than in the CT group. We and other investigators previously reported significantly lower systemic antioxidant capacity in blood samples from patients with POAG compared with controls^17–27^. Collectively, systemic changes in the oxidation/reduction balance were additionally confirmed in patients with glaucoma. The most important observation we made was that the serum levels of 9- and 13-(Z,E)-HODEs/LA in the POAG group were much higher than those in the CT group whereas the levels of 9- and 13-(E,E)-HODEs did not show the same results. These observations regarding the associations between 9- and 13-(Z,E)-HODEs and glaucoma were further confirmed in multivariate analyses (Table 4). These observations allow us to speculate on oxidation mechanisms in glaucoma subjects; that is, the significant difference between the POAG and CT groups was attributed to the difference in the amount of enzymatic oxidation. The down-regulation of activities in antioxidant enzymes such as glutathione peroxidase, superoxide dismutase, and catalase was reported in blood samples from patients with glaucoma^28–30^. Enzymatic oxidation is usually observed by an adaptive response *in vivo*^6^; thus, the down-regulation of antioxidant enzymes and/or the up-regulations of oxidant enzymes should be associated with higher levels of 9- and 13-(Z,E)-HODEs/LA in the POAG group in our study. In our previous comparative study between CT and POAG groups, we did not find any significant difference in serum levels of lipid peroxides that were measured by diacron reactive oxygen metabolites^17^, suggesting the possible specific involvement of these fatty acid metabolites (i.e., HODEs/HETEs) in the pathogenesis of glaucoma.

The differences in HODEs/HETEs levels between the CT and POAG groups were observed more remarkably in the HTG group than in the NTG group (Table 2). In multiple regression analyses, previous evidence suggested that oxidative stress was involved in glaucoma pathogenesis via its effects on IOP elevation in HTG and via its effects on RGC loss in POAG (i.e., HTG and NTG)^18–24^. We previously reported a significant correlation between lower systemic antioxidant levels and higher IOPs in subjects with glaucoma^27^. Collectively, the current results suggest that increased systemic oxidative stress markers are related more closely to IOP elevation than to neuronal damage. Interestingly, the serum levels of 10- and 12-(Z,E)-HODEs/LA were strongly correlated with the IOP (Table 3), which is one of the indexes of glaucoma severity that originates in TM cell dysfunction. One possible way of yielding singlet oxygen is by Type II photooxidation when a sensitizer is present in the vicinity of the reaction milieu, like in a cataract related to the photosensitization of tryptophan residues of human γD-crystallin^31^. The specific sites of oxidation have not been determined since sunlight may not reach TM cells Thus, the excretion and circulation of HODE formed in the eyes have not been determined yet; however, our previous observation suggested that HODE levels in the brain and spinal cord were reflected well by plasma levels^32,33^. Other studies have tested the statuses of systemic and local redox simultaneously and suggested that changes in systemic oxidant and antioxidant levels can reflect the local redox status^26,34,35^. We have reported previously that the plasma levels of 10- and 12-HODEs correlated significantly with a risk index for impaired glucose tolerance and insulin resistance and that they can be useful tools as biomarkers to detect early-stage diabetes^7,8,36^. In this case, we speculate that they are produced *in vivo* by the reaction between hydrogen peroxide and hypochlorite derived from myeloperoxidase. The latter is secreted by activated phagocytes^37,38^ or by eosinophils through a peroxidase-catalysed mechanism^39,40^. The significance of the possible up-regulation of 10- and 12-(Z,E)-HODEs in POAG disappeared in multivariate analyses (Table 4), suggesting that these might be specific markers of IOP elevation rather than the markers of glaucoma itself, although this hypothesis requires further assessment.

It was shown that evidence-based biomarkers were useful for proposing the effectiveness of antioxidants and other functional factors for the prevention of diseases^41,42^. Together with previous work, the present study may lead to the elucidation of oxidant/antioxidant statuses *in vivo* and to the proposal of a protective effect against glaucoma^15,17^.

## Methods

### Materials

Lipid-peroxidation products such as 8-iso-prostaglandin F_2α_ (isoP), isoP-d_4_, 5-hydroxyeicosa-6*E*,8*Z*,11*Z*,14*Z*-tetraenoic acid (5-HETE), 12-hydroxyeicosa-5*Z*,8*Z*,10*E*,14*Z*-tetraenoic acid (12-HETE), and 15- hydroxyeicosa-5*Z*,8*Z*,11*Z*,13*E*-tetraenoic acid (15-HETE) were obtained from Cayman Chemical Company (Ann Arbor, MI, USA); 13-hydroxy-9*Z*,11*E*-octadecadienoic acid (13-Z,E-HODE), 9-hydroxy-10*E*,12*Z*-octadecadienoic acid (9-E,Z-HODE), 13-hydroxy-9*E*,11*E*-octadecadienoic acid (13-E,E-HODE), 9-hydroxy-10*E*,12*E*-octadecadienoic acid (9-E,E-HODE), 10-hydroxy-8*E*,12*Z*-octadecadienoic acid (10-Z,E-HODE), 12-hydroxy-9*Z*,13*E*-octadecadienoic acid (12-Z,E-HODE), and 13S-hydroxy-10*E*,12*Z*-octadecadienoic-9,10,12,13-d4 acid (13-HODE-d_4_) were obtained from Larodan Fine Chemicals (Malmo, Sweden); and LA and AA were obtained from Sigma-Aldrich (St. Louis, MO, USA). Other materials were of the highest commercially available grade.

### Subjects

A total of 317 Japanese subjects comprised the POAG (n = 198) and non-glaucomatous CT (n = 119) groups. These subjects were recruited consecutively at Shimane University Hospital and Iinan Hospital, Shimane, Japan as described previously^17,25,27^. All measurements of oxidative stress markers were conducted at the National Institute of Advanced Industrial Science and Technology. The current study adhered to the tenets of the Declaration of Helsinki. The institutional review boards of Shimane University Hospital, Iinan Hospital, and the National Institute of Advanced Industrial Science and Technology reviewed and approved the research. All subjects provided written informed consent. All subjects underwent ophthalmologic examinations including measurements of best-corrected visual acuity (VA) and IOP by Goldmann applanation tonometry and slit-lamp, gonioscopic, and funduscopic examinations under pupillary dilatation. POAG was diagnosed based on the presence of an open iridocorneal angle bilaterally, the characteristic appearance of glaucomatous optic neuropathy such as enlargement of the optic disc cup or focal thinning of the neuroretinal rim, corresponding visual field defects identified using the Humphrey Visual Field Analyzer (Carl Zeiss Meditec, Dublin, CA) in at least one eye, and no evidence of secondary glaucoma bilaterally. In the POAG group, 114 subjects with a history of an untreated IOP of 21 mmHg or higher in at least one eye were considered to have HTG and the other 84 subjects who did not have a history of an untreated IOP of 21 mmHg or higher were considered to have NTG. The CT subjects had a corrected VA of 0.7 or better measured in both eyes using a decimal VA chart, and no glaucomatous optic neuropathy or history of an IOP of 21 mmHg or higher. Except for a cataract and/or glaucoma, no subjects had ocular pathologies such as clinically detectable ocular inflammation, infection, neuropathies, retinopathies, or maculopathies.

### Recording clinical parameters and blood sample processing

To avoid the possible confounding effect of systemic diseases, the subjects were questioned about their histories of severe systemic diseases during interviews before enrolment in the study. These diseases included acute brain infarction and haemorrhage, systemic neurologic diseases, cardiac diseases requiring catheter placement or surgery, cardiac failure and other systemic diseases affecting the subjects’ physical activity, lung diseases causing dyspnoea, chronic and acute hepatitis requiring interferon therapy, liver cirrhosis, renal failure requiring haemodialysis, autoimmune diseases requiring systemic steroids and other immunosuppressive therapies, severe anaemia requiring blood transfusions, major visceral surgery, malignancies, severe hypertension causing cardiac and kidney failure, and severe diabetes requiring insulin therapy. In addition to a history of severe systemic diseases, to adjust for the possible confounding effects of other factors such as differences in nutrition, blood pressure, blood glucose, and smoking habits, the presence or absence of diabetes, current smoking status, and time since the last meal, systolic blood pressure (SBP), DBP, and pulse rate were recorded before blood samples were collected. The highest known IOP measured on the day of sample collection or the highest previously measured IOP recorded in the medical charts also was collected. Venous blood specimens were collected from the antecubital vein into evacuated tubes. Serum samples obtained by centrifugation of the collected venous blood were stored at −80 °C until analysis. During all handling procedures, including transportation from the clinical setting to the laboratory and centrifugation, the temperature was maintained at 4 °C.

### Analysis of oxidative stress markers

The levels of HODEs, HETEs, and isoP were measured as described previously^3^ with slight modifications. The parent molecules—i.e., LAs and AAs—were detected using the same protocol. Briefly, 200 μL of plasma was mixed with 300 μL of saline and 500 μL of methanol containing the internal standards 8-iso-PGF_2α_-d_4_ and 13-HODE-d_4_, and 100 μM of butylated hydroxytoluene was added to the samples. This was followed by hydroperoxide reduction using an excess of triphenylphosphine and saponification with 1 M KOH in methanol. The mixture was acidified with 10% acetic acid in water and extracted with chloroform and ethyl acetate. An aliquot of the sample was analysed by liquid chromatography/tandem mass spectrometry (LC-MS/MS) on a TSQ Quantum Access Max system (Thermo Fisher Scientific, Waltham, MA, USA). The Dionex Ultimate 3000 system (Thermo Fisher Scientific) used for HPLC consisted of an HPG-3400 RS pump, WPS-3000 TPL RS Well Plate autosampler, and TCC-3000 RS column compartment equipped with a Hypersil Gold ODS column (3.0 μm, 100 × 2.1 mm; Thermo Fisher Scientific) set at 40 °C. Elution was performed using a gradient of solvent A (2 mM ammonium acetate in water) and solvent B (methanol:acetonitrile = 5:95) at a flow rate of 0.2 mL/min. The initial gradient composition was 80% A and 20% B. The composition was changed to 79% A and 21% B for 10 min and then to 0% A and 100% B for 45 min. Electrospray ionization was performed at a needle voltage of 3.0 kV. Nitrogen was used as the sheath gas (50 psi) and auxiliary gas (10 units). The capillary was heated to 300 °C and the mass spectrometer was optimized for maximum sensitivity. A specific precursor-to-product ion transition was achieved by monitoring selected reactions after collision-induced dissociation in negative mode. Argon was used as the collision gas, and the collision cell pressure was 1.5 mTorr. The precursor, product ions, and collision energy were determined after MS/MS optimization as follows: m/z = 353.1 and 193.1 at 26 eV for 8-iso-PGF_2α_; m/z = 357.1 and 197 at 26 eV for 8-iso-PGF_2α_-d_4_; m/z = 295.0 and 195 at 18 eV for both 13-Z,E-HODE and 13-E,E-HODE; m/z = 295.0 and 171 at 18 eV for both 9-E,Z-HODE and 9-E,E-HODE; m/z = 295.0 and 183 at 18 eV for both 10-Z,E-HODE and 12-Z,E-HODE; m/z = 299.0 and 198 at 18 eV for 13-HODE-d_4_; m/z = 319.0 and 115 at 14 eV for 5-HETE; m/z = 319.0 and 163 at 14 eV for 12-HETE; m/z = 319.0 and 203 at 14 eV for 15-HETE; m/z = 279 at 5 eV for LA; and m/z = 303 and 259 at 12 eV for AA.

### Statistical methods

All statistical analyses were performed using IBM SPSS Statistics version 21.0. (IBM Corp., Armonk, NY, USA). All continuous data are expressed as the mean ± standard deviation. For comparisons between the CT and POAG groups, the Mann-Whitney U test was used for continuous variables including age, SBP, DBP, pulse rate, time from the last meal, IOP on the blood sampling day, highest IOP recorded, and various oxidative stress makers (HODEs, HETEs). Fisher’s exact probability test was used for categorical variables including sex, diabetes status, and current smoking habits. For comparisons between the CT, NTG, and HTG groups, the Kruskal-Wallis test followed by the post hoc Mann-Whitney U test were used for continuous variables, and the G-test followed by the post hoc Fisher’s exact probability test were used for categorical variables. Possible correlations between demographic data and various oxidative stress markers were assessed by Spearman’s rank correlation test. To adjust for differences in demographic data between the groups, associations between glaucoma and oxidative stress markers were tested using multiple logistic regression analyses with CT/POAG set as dependent variables and six demographic factors (age, sex, DBP, diabetes, IOP on the blood sampling day, and the highest IOP recorded) and each of the oxidative stress makers (HODEs, HETEs) were set as independent variables. P-values of 0.0167 and 0.0033 were considered significant at levels of 5% and 1%, respectively, by the Bonferroni correction for three-group comparisons; otherwise, a p-value of 0.05 was considered statistically significant.
